# Interplay Between Aging and Tau Pathology in Alzheimer’s Disease: Mechanisms and Translational Perspectives

**DOI:** 10.3390/antiox14070774

**Published:** 2025-06-24

**Authors:** Mohammed Alrouji, Mohammed S. Alshammari, Syed Tasqeeruddin, Anas Shamsi

**Affiliations:** 1 Department of Medical Laboratories, College of Applied Medical Sciences, Shaqra University, Shaqra 11961, Saudi Arabia; malrouji@su.edu.sa; 2 Department of Clinical Laboratory Sciences, College of Applied Medical Sciences, Shaqra University, Shaqra 11961, Saudi Arabia; m.alshammari@su.edu.sa; 3 Department of Pharmaceutical Chemistry, College of Pharmacy, King Khalid University, Abha 62521, Saudi Arabia; hsyed@kku.edu.sa; 4 Centre of Medical and Bio-Allied Health Sciences Research, Ajman University, Ajman P.O. Box 346, United Arab Emirates

**Keywords:** Alzheimer’s disease, aging, tau pathology, tau phosphorylation, amyloid-β, neuroinflammation, oxidative stress

## Abstract

Aging is a key risk factor for neurodegenerative disorders and is associated with widespread systemic and brain-specific changes. Alzheimer’s disease (AD), a progressive and irreversible brain disorder, primarily affects older adults and leads to a gradual decline in cognitive function. The underlying disease mechanisms often begin years before clinical symptoms appear, limiting the effectiveness of current treatments. Several factors linked to aging—including inflammation, oxidative stress, impaired metabolism, and protein aggregation—contribute to the onset and progression of AD. A central feature of AD is the abnormal accumulation of amyloid beta (Aβ) and tau, a microtubule-associated protein, driven by post-translational modifications such as acetylation and hyperphosphorylation. These modifications lead to structural changes in tau, promoting the formation of neurofibrillary tangles (NFTs), which are more closely associated with cognitive decline than Aβ plaques. Interestingly, tau accumulation and the resulting cognitive impairments are often observed in aged individuals without Aβ deposition, highlighting tauopathy as a distinct contributor to age-related cognitive decline. This review focuses on new developments in therapeutic approaches that target oxidative stress, protein aggregation, and neuroinflammation, and our current understanding of the molecular pathways relating aging and tau pathology in AD.

## 1. Introduction

Age-related illnesses, particularly Alzheimer’s disease (AD), have become a major public health concern as a result of the world’s population rapidly aging. One of the main causes of dementia in the elderly is AD, a neurodegenerative disease [1]. According to a report published in 2025, an estimated 57 million people worldwide had dementia in 2021, with AD accounting for 60–70% of cases [1]. As people age, the prevalence of AD rises, particularly after the age of 65. Age is therefore regarded as the main risk factor for AD. Nevertheless, it is still unknown what mechanisms underlie the age-related vulnerability to AD [2]. First proposed in 2013, the hallmarks of aging are defined based on three premises: they should be associated with age, they can be accelerated by experimental manipulation, and they can be stopped or reversed through therapeutic intervention [3].

The term “healthy brain aging” describes aberrant aging characteristics within the body’s manageable range and does not have detrimental effects. On the other hand, “pathological brain aging” describes the occurrence of aberrant aging characteristics outside of the body’s controllable range. In addition to aging, the abnormal buildup of amyloid beta (Aβ) or neurofibrillary tangles (NFTs) in the brain causes the transition from early (prodromal) to established AD. Numerous brain cell types in AD patients and experimental models have shown signs of cellular senescence, which builds up and causes neurodegeneration and age-related disorders [4]. Removing senescent cells and other signs of aging may improve cognition and reduces tau- and Aβ-induced neuropathology in AD rats [5,6,7], indicating that aging is a key risk factor in the pathophysiology of AD. Nevertheless, much more needs to be explored about the neuro-pathophysiological function of aging and its underlying mechanisms in AD. Researchers are working to advance the therapeutic options for this disease [8,9,10].

Aging is a biological process that leads to a cumulative and irreversible loss of function across all organ systems and these changes occur due to the buildup of damage in response to a variety of stressors [11]. Physical degeneration that comes with aging raises the chance of illness and death [12]. Aging is marked by organ damage, metabolic decline, bone loss, inflammation, and cellular aging—driven by molecular changes in nutrient sensing, protein balance, mitochondrial function, and DNA repair pathways [13]. These changes can be worsened by a variety of external factors ranging from chemotherapy to environmental factors [14]. Different species age at different rates, and there are also inter-individual differences within a species and between an individual’s different tissues [15]. Each of these factors raises the chance of developing age-related illnesses. Research has shown nine essential characteristics of the aging process, and potential biomarkers of aging are consistent with the main molecular mechanisms of aging. The aging process has by far the most significant influence among the several risk factors for neurodegeneration. Therefore, it is critical to take into account the fundamental mechanisms of aging and how they contribute to the development and progression of neurodegenerative illness to create effective therapies.

Given its significant impact on life expectancy and quality of life, neurodegeneration and the resulting cognitive loss are among the most significant age-related disorders. Due to the high frequency of neurodegenerative diseases and the rarity of brains in elderly individuals that are completely free from pathological changes—such as amyloid plaques, tau tangles, or other neurodegenerative markers—brain aging is increasingly seen as a continuum with neurodegeneration. Neurodegenerative disease progression is shaped by both genetic and environmental factors [16]. According to some researchers, aging and AD may lie on a pathological spectrum, although advanced age is the greatest risk factor for AD. The mechanisms underlying AD and normal brain aging are still not fully understood, and the data points to AD as a unique neurodegenerative disease rather than merely an accelerated type of aging [2,17]. The risk of AD increases with age, making elderly individuals more susceptible [18]. It is unclear whether the levels of aggregated proteins like Aβ, α-synuclein, and hyperphosphorylated tau (p-tau) are associated with the severity of the cognitive impairment, despite molecular studies showing that older people’s brain tissue contains abnormal deposits of these proteins [19]. According to specific research, early developmental problems may increase the likelihood of neurodegenerative disease, suggesting that structural alterations in the brain may occur much earlier than the cognitive impairment [20]. It has been suggested that exposure to harmful environmental stimuli during development, such as drugs, trauma, or environmental pollutants, may impact neuroplasticity later in life [21]. This review highlights the key molecular pathways in neurodegeneration and brain aging, along with potential therapeutic strategies.

## 2. Aging and the AD Brain

**Figure 1:**
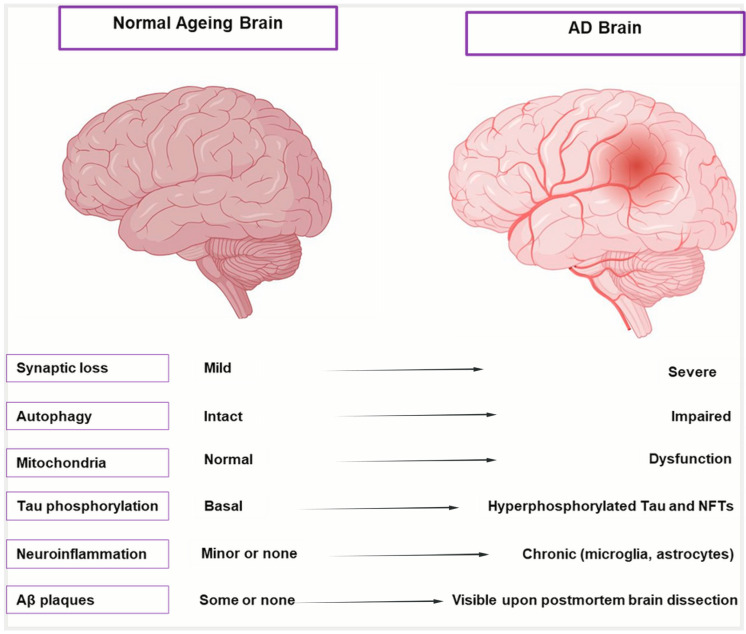
Cellular and molecular hallmarks of normal aging vs. AD brain. Illustration comparing a healthy aging brain (**left**) with an AD-affected brain (**right**). The AD brain shows significant shrinkage of the cortex and hippocampus, enlarged ventricles, and widened sulci due to neuronal loss. Key AD pathologies—amyloid-β plaques and neurofibrillary tangles—are also depicted, distinguishing it from the relatively intact structure of the normal aging brain.

Aging initiates a complex cascade of biological changes that significantly increase the brain’s vulnerability to AD, beyond merely being a risk factor [22]. Aging causes distinct anatomical, cellular, and molecular alterations in the brain, as shown in Figure 1 and described in Table 1, which highlight the transition from healthy aging to AD. These alterations include moderate shrinking of brain areas, low-level neurodegeneration, and subtle cognitive loss. These alterations are intensified in AD, leading to severe cognitive impairment, synaptic dysfunction, extensive neuronal death, and hippocampus atrophy. To differentiate between the early phases of neurodegeneration and healthy aging, it is crucial to comprehend this spectrum. Despite significant efforts to combat this illness, AD is still incurable and unstoppable and patients are frequently diagnosed at a late and irreversible stage, facing an average survival period of 4–8 years [23]. It was believed that our limited understanding of the intricate pathogenic mechanism of AD was the primary cause of the high failure rate of medication development [24]. Many different factors influence the prognosis of AD. The intricacy of AD is revealed by the various theories on its underlying origin. Several factors have been suggested to contribute to the development of AD, including cholinergic Aβ toxicity, tau protein hyperphosphorylation, oxidative stress, and neuroinflammation [25,26]. All these factors accelerate the disease’s course, regardless of the underlying cause of AD.

## 3. Pathological Mechanism of AD

AD is a progressive neurodegenerative disease that results in brain atrophy, death, and the loss of neurons and synapses. Disappointing progress has been made in the fight against AD on the fronts of prevention and cure, despite massive basic and clinical research and multibillion-dollar investments [24,43]. In the absence of viable treatments, AD threatens to spread around the world.

Genetic risk factors for AD include mutations in presenilin-1, presenilin-2, and the amyloid precursor protein (APP) gene and these mutations are primarily linked with familial, early-onset forms of AD [44]. The frequency of sporadic (late-onset) AD is significantly higher and is believed to result from the intricate interplay between environmental and genetic variables. The most prevalent genetic risk factor for sporadic AD is the APOE*ε4 allele [44].

Aβ plaques and p-tau NFTs in the brain are two of AD’s most noticeable molecular characteristics [45,46]. Aβ is a small aberrant proteolytic fragment created when β-secretase and γ-secretase sequentially cleave APP [45]. Tau and Aβ have emerged as key targets for AD therapy research because of the typical histopathological features of AD, which include abnormal fibrillar deposits (plaques) of Aβ peptide and NFTs of tau protein [47,48]. Neuronal loss is associated with Aβ plaques, oligomers, and fibrils that build up in the brain [49]. APP, a bitopic integral protein in neuronal membranes, is proteolyzed to produce Aβ, a peptide of variable length (usually 42 or 40 amino acids, called Aβ 42 and Aβ 40 [45]. The amyloid cascade theory [50], which links the buildup of aggregated Aβ to AD, was prompted by the discovery of Aβ plaques in AD brains. Numerous in vitro and in vivo investigations have reported the cytotoxicity of Aβ, with prefibrillar Aβ oligomers being identified as the main hazardous species [51,52]. Between 10 and 50% of the total Aβ in AD brains are N-terminally truncated and pyroglutamylated (at Glu3 or Glu11) Aβ peptides (pEAβ), which have been demonstrated to have increased cytotoxicity [53,54]. Furthermore, early-onset AD, which affects individuals under 60 and accounts for around 5% of all AD cases, is linked to a group of pathogenic mutations in the PSEN1, PSEN2, and APP genes, including changes in the Aβ-encoding sequence [55].

Another possible cause of AD is thought to be p-tau, which builds up in neurons in AD and results in neuronal dysfunction [56,57]. Tau is a microtubule-associated phosphorylatable protein that is crucial for axonal transport and maintains the integrity and functionality of neurons’ tubular cytoskeleton [58,59]. Dysregulation of microtubule dynamics, synaptic failure, and neuronal dysfunction result from the misfolding, aggregation, and development of neurofibrillary tangles of p-tau [60,61]. The degree of neurodegeneration and the course of the disease are correlated with the slow buildup of phosphorylated tau at particular locations in AD brains [62,63]. Given that tau phosphorylation is known to be mediated by Aβ monomers and oligomers, the Aβ and tau protein neurotoxicity routes in AD are probably connected [64,65,66,67].

Both tau and Aβ diffuse across the brain in distinctive ways as AD progresses. It has been demonstrated that non-neuronal brain cells, such as oligodendrocytes, astrocytes, and microglia, contribute to the spread of tau and Aβ in the brains of transgenic animal AD models [68,69]. Both tau and Aβ can be transferred into the peripheral bloodstream by brain exosomes, which can penetrate the blood–brain barrier (BBB) [70].

One of the main causes of AD is the neuroinflammatory response, an immunological reaction in the brain brought on by internal or external stress, excessive oxidation, and the buildup of Aβ plaques or p-tau. This response is primarily driven by aberrant activation of astrocytes and microglia, which release pro-inflammatory cytokines. DNA damage and mitochondrial malfunction may also be risk factors for AD. In AD patients, telomere maintenance defects are linked to amyloid pathology, cognitive decline, and tau hyperphosphorylation through oxidative stress and inflammation [3,71].

The FDA has approved acetylcholinesterase inhibitors (donepezil, galantamine, and rivastigmine) for the treatment of AD, but they only partially reduce symptoms and have little effect on stopping the disease’s progression [72]. The FDA gave accelerated approval to Aducanumab as a treatment for AD in 2021 but was discontinued by its manufacturer (Biogen) in 2024 [73]. In animals and humans, telomere maintenance defects hasten aging; in AD patients, they are linked to amyloid pathology, cognitive decline, and tau hyperphosphorylation through oxidative stress and inflammation [3,71].

Research has demonstrated that protein aggregation happens when soluble protein monomers combine to form polymers, high-molecular-weight oligomers, and ultimately massive insoluble fibrils [71]. Although it is unknown what the biological mechanisms are, these protein clumps reduce neuronal activity. There is growing evidence that AD is associated with impaired mitophagy, which leads to the buildup of defective mitochondria. Cellular senescence, another characteristic of aging, makes people more vulnerable to AD, PD, and other neurological diseases [11,74].

## 4. Oxidative Stress and AD

**Figure 2:**
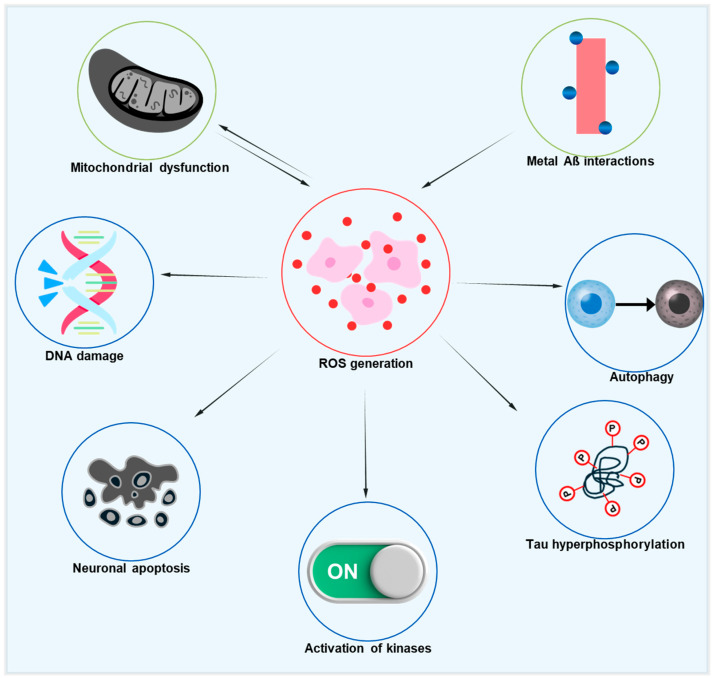
Diagrammatic illustration of the pathogenic cascades in AD initiated by ROS. Excessive ROS production, driven by mitochondrial dysfunction and metal–Aβ interactions, leads to DNA damage, impaired autophagy, tau hyperphosphorylation, the activation of pro-pathogenic kinases, and neuronal apoptosis. Notably, a reciprocal link between mitochondrial dysfunction and ROS generation creates a vicious cycle that accelerates neurodegeneration.

Improving mitochondrial activity can significantly impact systemic age-associated oxidative stress, as mitochondrial failure is common in aging. Thus, targeting stressed and damaged mitochondria may be a key strategy for reducing oxidative stress in AD models. The relationship between oxidative stress, mitochondrial dysfunction, and AD pathology is represented in Figure 2, which also depicts the downstream effects of oxidative damage on tau phosphorylation and neuronal degeneration. This diagram illustrates how excessive ROS formation, arising from dysfunctional mitochondria and metal–Aβ interactions, starts a chain reaction that damages DNA, impairs autophagy, causes tau hyperphosphorylation, activates kinases like GSK-3β and CDK5, and eventually results in neuronal degeneration. Additionally, mitochondrial dysfunction and ROS are linked in both directions, creating a vicious cycle that speeds up AD’s neurodegenerative processes [75]. Significant deficiencies in mitochondrial metabolism, particularly a decrease in the α subunit of the mitochondrial F1 ATP synthase, which connects oxidative phosphorylation to ATP generation, are seen in healthy aging. This results in higher oxidation of DNA, proteins, and lipids, decreased ATP synthesis, and increased formation of ROS [76,77,78]. In addition to mtDNA damage, mitochondrial dysfunction also causes nuclear DNA damage, especially in the promoter region of age-downregulated genes related to mitochondrial, vesicular, and synaptic plasticity function [79]. Although antioxidant overexpression has been investigated as a potential strategy to counteract the negative effects of aging, it has not been shown to increase longevity, except in flies that overexpress superoxide dismutase (SOD).

Since elevated mtDNA oxidation is one of the initial indicators of AD, dysfunctional mitochondria are also linked to the disease’s pathophysiology. Indeed, age-associated mitochondrial decrease may be one of the earliest steps in the pathophysiology of sporadic, late-onset AD [80,81,82]. According to the mitochondrial cascade hypothesis, APP processing and expression are impacted by age-related mitochondrial dysfunction. This results in Aβ oligomers that build up into plaques in AD [83,84]. It was demonstrated that neuronal toxicity is caused by the hydrophobic 25–35 region of Aβ, producing ROS, proving that Aβ is a potent oxidative stressor in and of itself [85,86]. Aβ interactions with redox-active metals are probably what cause the oxidative damage. Aβ is bound by copper, zinc, and iron, encouraging the protein to aggregate into plaques. It has been demonstrated that this combination produces hydrogen peroxide and superoxide, with copper forming the most stable link among these metals [87,88,89]. Metal–amyloid complexes induce oxidative stress that damages mitochondrial function, causes excitotoxicity, and encourages membrane depolarization. Impaired insulin synthesis, glucose metabolism, and mitophagy contribute to AD brain mitochondrial dysfunction. Patients with moderate cognitive impairment (MCI), a pre-clinical stage of AD, have decreased brain glucose metabolism before the disease manifests [90].

Recent research has demonstrated that the pathophysiology of AD can be attenuated by reducing the harmful consequences of mitochondrial dysfunction. In 3xTg mice, overexpression of the mitophagy regulator parkin restored neurotransmitter production while reducing Aβ buildup and damaged mitochondria [91]. Thus, targeting stressed and damaged mitochondria may be a key strategy for reducing oxidative stress in AD models.

## 5. Tau Pathology in AD

Tau deposition is also common in the aged brain, specifically in age-related tauopathy [92,93]. According to clinical evidence, tauopathies associated with aging may be connected to other neurodegenerative conditions linked to NFT development [94,95,96]. Even though age-related tauopathies are commonly observed in older patients’ brains and are usually detectable after death, diagnosing them is still challenging. Clinically, tauopathies related to aging may be linked to or correlated with other neurodegenerative diseases, such as NFT formation and senile plaques [97]. Region-specific patterns of tau pathology, especially in the hippocampus and neocortex, have been identified through recent tau immunohistochemistry investigations in amnestic and non-amnestic AD variations. This implies that tau-mediated neurodegeneration may follow conserved anatomical pathways, and this might extend to Parkinson’s disease (PD), where overlapping tau involvement is seen. This is corroborated by the differing vulnerability and distribution of phosphorylated and cleaved tau species [98]. It is interesting to note that there is growing evidence that PD and AD share tau pathology, especially in the medial temporal lobe and other relevant brain regions. Tau buildup is seen in both conditions, and these similarities raise the prospect of convergent neurodegenerative processes. Finding more general treatment targets that cut across disease borders may be made easier with an understanding of the tau-related alterations that are shared by AD and PD [99]. However, the prospect of employing tau imaging as a diagnostic tool to identify age-related tauopathy in clinical settings will only be made possible by discovering the correlation between tauopathy and age-related tauopathy [97,100,101].

**Figure 3:**
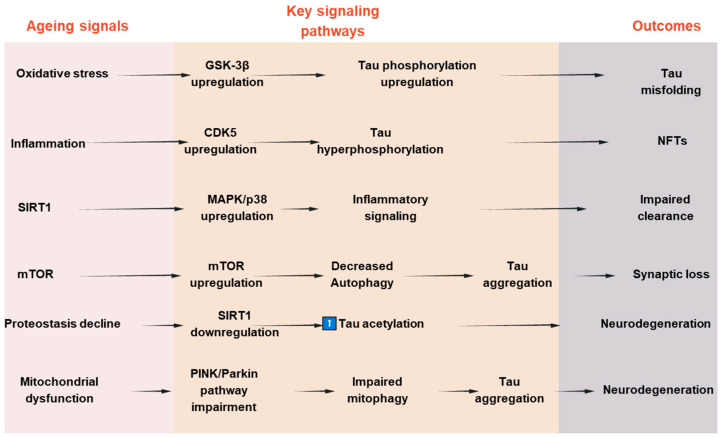
Signaling pathways involved in tau pathologies and aging-associated neurodegeneration.

**Figure 4:**
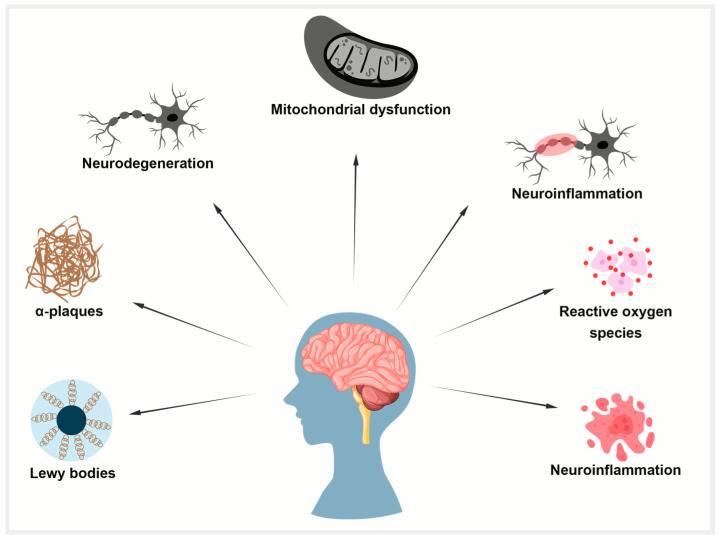
Common mechanisms in aging, AD, and PD. Aging-related mitochondrial dysfunction, oxidative stress, and impaired protein clearance promote tau pathology. In AD, tau forms neurofibrillary tangles; in PD, it co-aggregates with alpha-synuclein in Lewy bodies.

To establish a connection between tau deposition and common aging mechanisms, numerous attempts have been made to clarify the underlying molecular mechanism. Among these, it has been demonstrated that aging is significantly impacted by changes in the kinase activity of the mammalian target of rapamycin (mTOR) [102,103]. It has been demonstrated that mTOR inhibition increases autophagy, which in turn helps lessen tauopathies and protein aggregation in neurodegenerative conditions [104,105]. Second, tau accumulation is linked to aging in the healthy aging brain due to a reduction in the activity of the proteasomal degradation machinery, which results in undesirable protein aggregates [106]. Third, aging causes the protein deacetylase SIRT1 to become downregulated or inactive. SIRT1 is necessary to limit oxidative stress and maintain the levels of neurotrophic factors like BDNF to preserve brain function [107,108,109,110]. When SIRT1 is inactivated in AD, tau acetylation increases, a prelude to tau phosphorylation and tauopathy. Tau pathology in AD is caused by changes in signaling pathways such the mTOR, GSK-3β, CDK5, MAPKs, and SIRT1 pathways, which cumulatively influence tau phosphorylation, aggregation, and clearance. Their dual relevance to tauopathy and age-related neurodegeneration is highlighted by the fact that these pathways not only regulate tau dynamics but also mimic more general aging-associated cellular stress responses. A schematic summary of these pathways and their interactions in tau pathology is presented in Figure 3. To further illustrate the convergence of tau-related mechanisms in aging, AD, and PD, a new integrative schematic is depicted in Figure 4. Although the primary focus of the review is on AD, we have included PD to highlight the overlapping pathogenic pathways—particularly mitochondrial dysfunction, oxidative stress, and impaired proteostasis—that contribute to tau pathology in both disorders. Finding more general treatment targets that cut across disease borders may be made easier with an understanding of the tau-related alterations that are shared by AD and PD.

### 5.1. Tau Pathology Beyond AD

Although tau pathology is a well-known feature of AD and primary tauopathies, such as corticobasal degeneration (CBD) and progressive supranuclear palsy (PSP), its association with PD is becoming more widely acknowledged. It is well established that tau plays a key role in the pathophysiology of AD and progressive supranuclear palsy (PSP) but in recent times, there has been growing evidence that points to the involvement of tau in the pathophysiology of PD [111].

According to a study by, tau aggregation is observed in nearly half of PD brains, with evidence implying that it spreads from one neuron to another. The buildup of neurofibrillary tangles (NFTs) and abnormal hyperphosphorylated tau, and tau’s interaction with alpha-synuclein may together drive neuronal death and disrupt axonal transport, thereby contributing to the progression of PD and Parkinsonism [112]. Interestingly, tau frequently co-localizes with alpha-synuclein in Lewy bodies, demonstrating that tau and synucleinopathies share a common mechanism. This co-pathology may impact PD’s clinical variability and course, highlighting the possible applicability of tau-targeted treatments outside of AD. According to a recent study by Chu et al. [113], nigrostriatal degeneration (progressive loss of neurons in a specific brain pathway called the nigrostriatal pathway that is critical for movement control) can happen without alpha-synuclein pathology, according to the results from a cohort of people with modest motor impairments, a prodromal stage that does not fully fit the criteria for PD. Both groups in this work (with and without nigral alpha-synuclein aggregation) exhibited consistent buildup of phosphorylated tau (AT8, Ser208, Ser396/404) and similar losses of dopaminergic neurons and putamenal innervation. Interestingly, the age-matched controls did not have significant tau pathology. These findings imply that tau pathology, rather than synuclein aggregation, may be the primary cause of dopaminergic neurodegeneration in early PD.

Alpha-synuclein buildup in Lewy bodies has historically been thought to be the main cause of PD, which is classified as a synucleinopathy. Recent neuropathological research, however, has shown that in the early stages of PD, tau pathology may occur before or concurrently with alpha-synuclein pathology. Notably, even in the absence of Lewy body pathology, substantial tau accumulation and dopaminergic neuron loss were noted in people with moderate motor deficits (MMDs), a prodromal clinical state that does not fully fit the diagnostic criteria for Parkinson’s disease. These results cast doubt on the current alpha-synuclein-centered proteinopathy paradigm by indicating that tau may have an independent and perhaps initiating role in nigrostriatal degeneration. According to this viewpoint, PD is entering a “Tau-lemaic era,” in which tau pathology is being re-examined as a potential key factor in the early neurodegenerative processes of PD rather than as a subsequent occurrence.

### 5.2. Tau Phosphorylation in AD

Tau can be phosphorylated at 63 locations in WT and hAPP transgenic mice under normal conditions [114]. However, compared to a healthy brain, which has 2–3 mol P/mole of tau protein, the AD brain has 2–3 times more hyperphosphorylated tau (6–8 mol P/mole of tau protein) [115]. At least 40 serine/threonine and 2 tyrosine phosphorylation sites in the PHF version of tau were found using mass spectrometry analysis [116]. Subsequent research showed that the interaction of several kinases and phosphatases results in tau hyperphosphorylation. Numerous proline-directed protein kinases (PDPKs) primarily phosphorylate tau on serine/threonine residues preceded by a proline residue. Cyclin-dependent kinase-5 (CDK5), dual-specificity tyrosine-phosphorylation-regulated kinase 1A (DYRK1A), and glycogen synthase kinase-3beta (GSK-3beta) are important tau PDPKs [117,118,119,120]. Tau can also be phosphorylated by several non-PDPKs, including casein kinase 1 (CK1), microtubule affinity-regulated kinase 110 (MARK p110), calcium/calmodulin-activated protein kinase II (CaMK II), and protein kinase A (PKA) [121,122,123]. About 70% of the overall tau phosphatase activity in the central nervous system is attributed to PP2A, which primarily regulates the dephosphorylation of tau. Table 2 lists the tau-phosphorylation-related kinases. 

### 5.3. Tau Acetylation in AD Promotes Tau Phosphorylation

As a substantial regulatory post-translational modification linked to multiple tauopathies, including AD pathogenesis, tau acetylation has recently drawn particular attention [138,139]. Tau acetylation is connected to neurodegeneration and various tau acetylation sites, including potential sites Lys 163, Lys 173, and Lys 180, are known. These residues stop tau ubiquitination and, consequently, tau turnover. It was demonstrated that tau acetylation mediated by p300 and lysine acetyltransferase promotes phosphorylated tau aggregation, which may be prevented by deacetylating tau with SIRT1 deacetylase. Acetylation of the lysine 280 residue is significant for tau phosphorylation [138,140,141]. Lysine 174 is a novel acetylation site that is likewise acetylated by p300. Acetylation on lysine 174 is a powerful tau alteration that causes tau to aggregate and impair cognitive function [142]. Acetylated tau is connected to synaptic dysfunction and cognitive decline via AMPA receptor trafficking [143]. In addition to its involvement in neurofibrillary tangle production and microtubule instability, pathogenic tau harms mitochondria. Mitochondrial dysfunction has been suggested as an underlying mechanism of AD pathophysiology [144]. Besides disrupting mitochondrial dynamics by altering the balance of fission and fusion, hyperphosphorylated tau induces aberrant mitochondrial distribution within neurons, thereby impeding mitochondrial transport along axons [144]. This mislocalization increases the formation of reactive oxygen species (ROS) by restricting the flow of ATP to synaptically active areas. Moreover, tau buildup exacerbates mitochondrial dysfunction by interfering with complex I and calcium buffering, which are vital for mitochondrial function. These tau-induced changes reinforce the crucial role that mitochondrial dysfunction plays in AD pathophysiology by causing synaptic failure and neuronal death.

Several acetyltransferases and deacetylases have been reported to alter the degree of tau acetylation in AD conditions. It was previously stated that tau might be acetylated by p300 or CBP acetyltransferases and deacetylated by SIRT1 [140].

Conversely, a negative association between tau and SIRT1 has also been found. According to this study, tau pseudo-acetylation can reduce Aβ-mediated toxicity and stop tau from becoming phosphorylated [145]. However, more research is necessary to determine the missing mechanism linking Aβ to acetylated tau in AD conditions. In neurodegenerative diseases like AD, stress has been linked to synaptic loss and neuron death [146,147]. This is because nitrosylation of proteins occurs in these settings. According to this research, Aβ-induced NO generation nitrosylates GAPDH, which further acetylates tau protein by promoting p300 acetyltransferase activation and acetylation [148]. Nitrosylated GAPDH increases the likelihood of additional tau acetylation by nitrosylating the deacetylase, SIRT1.

## 6. Aβ in AD

The short peptide known as Aβ, which has 40–42 amino acids, is created when the proteolytic enzymes beta (β)-secretase and gamma (γ)-secretase cleave APP intracellularly [149]. APP is widely expressed in the brain and is found at neuronal synapses [150]. It has been connected to neuroprotection, cell–matrix or cell–cell interactions, synaptic plasticity, and the control of neuronal cell development [151]. Aβ is essential for synaptic structural–functional plasticity, the basis for learning and memory based on the enhanced long-term potentiation (LTP) mediated by Aβ40 [152].

**Figure 5:**
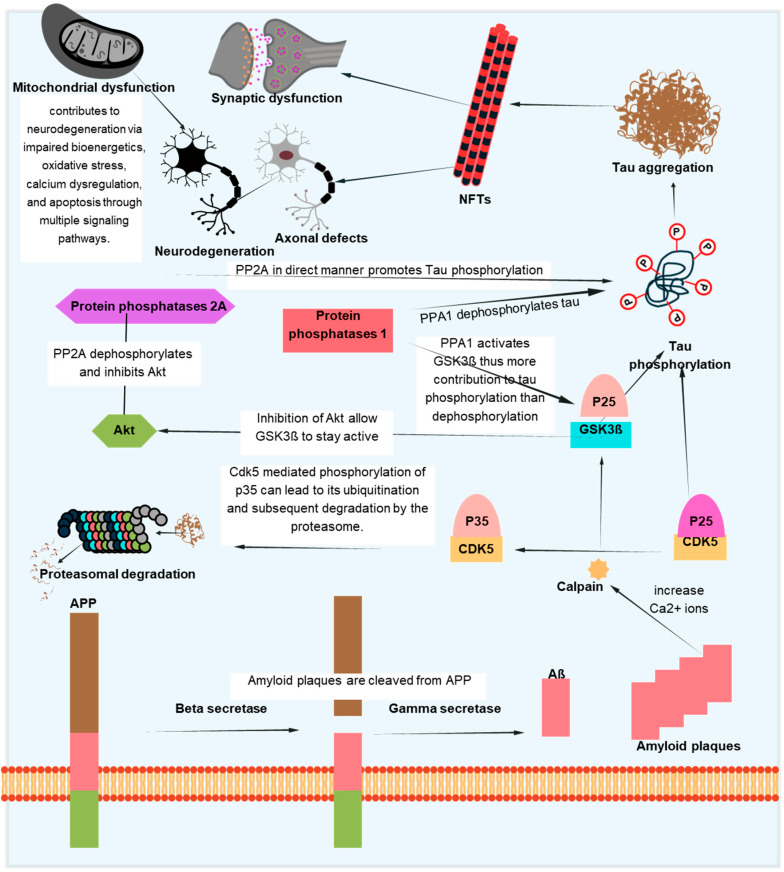
Pathological interactions between tau and Aβ in AD. This schematic illustrates one of the several proposed mechanisms linking Aβ accumulation to tau hyperphosphorylation and NFT formation. Aβ-induced calcium influx activates calpain, which cleaves p35 to p25, resulting in persistent CDK5 activation. Alongside GSK3β and DYRK1A, this contributes to tau phosphorylation and aggregation. The pathological cascade also involves mitochondrial dysfunction, synaptic damage, and axonal defects, ultimately promoting neurodegeneration. While this figure focuses on a specific pathway, other routes connecting Aβ and tau also exist.

The aggregation of the Aβ-peptide causes synaptic dysfunction and neurodegeneration, as explained by the amyloid hypothesis [153]. Errors in the systems controlling the generation, accumulation, or removal of Aβ are the primary cause of AD. Aβ aggregation disrupts cell-to-cell communication and triggers the immune system, which leads to inflammation and ultimately the death of brain cells. Another pathogenic cause of AD is the buildup of tau, which is hyperphosphorylated by Aβ aggregation and results in NFTs [154]. Tau supports the brain’s neuronal architecture and several other important functions under normal circumstances [155]. But, in pathological conditions, tau becomes hyperphosphorylated and clumps together to form fibrils called neurofibrillary tangles. Neurotoxicity and neuronal degeneration result from the buildup of aberrant tau and tangles in neurons [33,156]. Tau phosphorylation affects not just the production of NFTs but also tau’s capacity to bind microtubules, which affects neuronal functions like mitochondrial respiration and axonal transport [157,158,159]. Neuronal cell death results from tau hyperphosphorylation-induced microtubule depolymerization, self-aggregation, and separation [160]. Figure 5 depicts one of several mechanistic pathways through which Aβ accumulation promotes tau pathology in AD. Increased intracellular calcium levels brought on by the deposition of Aβ peptides, especially in oligomeric forms, activate the calcium-dependent protease calpain. A prolonged and hyperactive CDK5/p25 complex is created when calpain cleaves the CDK5 activator p35 into p25. In addition to the contributions of GSK3β and DYRK1A, this abnormal kinase activity causes tau to become excessively phosphorylated. After undergoing conformational changes, hyperphosphorylated tau separates from microtubules, clumps together to form paired helical filaments, and finally forms NFTs. This pathogenic cascade has a role in the death and the malfunctioning of neurons. Abnormal protein aggregation, mitochondrial dysfunction, decreased neurotransmitter production, inflammation, and oxidative stress all contribute to the entire process. Although Figure 5 focuses on one such process, tau pathology in AD is linked to Aβ buildup through a number of overlapping mechanisms, as discussed in [161,162].

## 7. Neuro-Inflammation in Aging and AD

Aging and neuroinflammation are closely related processes, with aging itself predisposing the brain to a persistent low-grade inflammatory state known as “inflammaging” [163,164]. Immunosenescence, which includes the dysregulation of both innate and adaptive immune responses, is a phenomenon that occurs with aging [165]. As people age, the major immune cells in the central nervous system (CNS), known as microglia, experience severe functional deficits that result in chronic neuroinflammation and prolonged activation [166]. Microglia improve Aβ clearance. However, persistent Aβ production makes microglia chronically active, which encourages further Aβ deposition [164,165], resulting in the generation of reactive oxygen species and neurotoxic pro-inflammatory cytokines [167]. This chronic state is further exacerbated by mitochondrial dysfunction, defective autophagy, and oxidative stress, which all contribute to an increase in production of pro-inflammatory cytokines, such as IL-6, TNF-α, and IL-1β [168,169]. The persistent activation of microglia results in the secretion of inflammatory mediators that can damage neurons, disrupt synaptic function, and impair cognitive processes [170,171].

Additionally, cellular senescence in astrocytes leads to the release of pro-inflammatory factors known as the senescence-associated secretory phenotype (SASP), further fueling neuroinflammation [172,173]. Tau hyperphosphorylation results from ROS-activating p38 mitogen-activated protein kinase (p38 MAPK) [174]. p38 MAPK has been linked to neuroinflammation and AD due to its ability to activate NF-κB in the brains of AD patients [175,176,177]. Systemic inflammation can exacerbate neuroinflammation in the aged brain due to the two-way connection between the brain and the peripheral immune system. Neuroinflammation is both a result of and a cause of brain aging because it is essential to the pathophysiology of neurodegenerative illnesses like AD [29,178,179]. Understanding the molecular mechanisms underlying this relationship may provide therapeutic opportunities to mitigate age-related cognitive decline and neurodegenerative diseases.

## 8. Therapeutic Interventions in Aging and AD: Molecular Targets and Translational Approaches

A key aspect of translational neurotherapeutics is understanding and influencing the molecular basis of tau pathology and the way it interacts with aging and other pathological features of AD. With an emphasis on substances currently undergoing clinical trials and testing, this section describes key therapeutic approaches that address tau pathology, Aβ interactions, mitochondrial dysfunction, and neurodegenerative pathways.

### 8.1. Tau-Targeted Therapeutics

**Figure 6:**
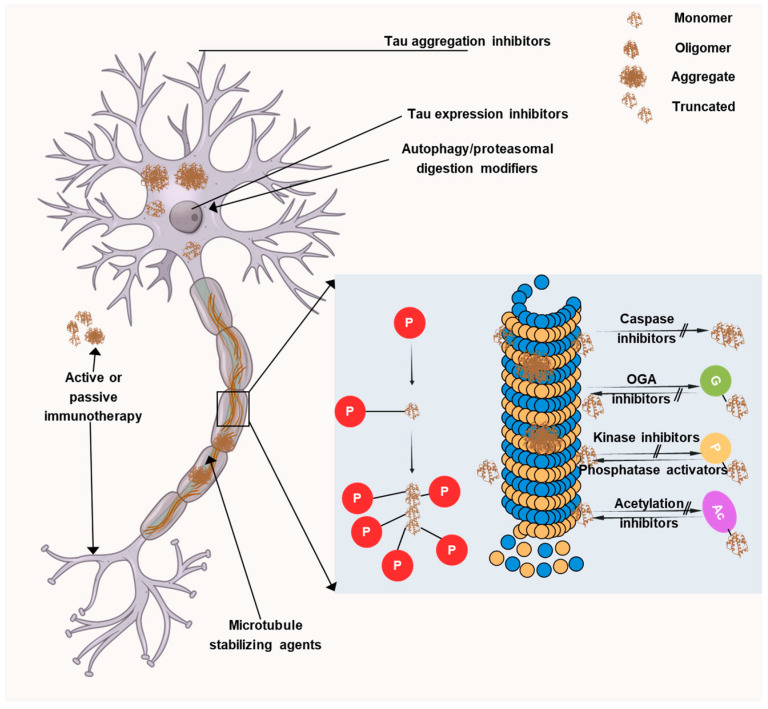
Therapeutic targets associated with tau. The image illustrates the many tau-targeting strategies being developed in preclinical or clinical settings to treat primary tauopathies andAD. LMTX, a derivative of methylene blue, and curcumin are inhibitors of tau aggregation. Tau’s typical microtubule-stabilizing action can be restored by using microtubule stabilizers like TPI-287 and NAP.

Current therapeutic approaches are mainly aimed at targeting Aβ plaques but despite many efforts, no significant results have been obtained. Recently, tau proteins have opened up a new avenue for developing novel AD therapeutics and thus, many researchers are focusing on it. Targeting tau is more likely to be effective after cognitive impairment and this is attributable to the fact that tau exhibits a better connection with symptom severity than Aβ. Initially, anti-tau therapies centered on microtubule stabilization, tau aggregation inhibition, and post-translational modifications. However, toxicity and/or ineffectiveness necessitated the end of many promising trials. Thus, recently, the focus has shifted toward immunotherapeutic approaches, with the majority of current clinical trials investigating monoclonal antibodies targeting various tau species. Four monoclonal antibodies have entered phase II clinical trials, namely, semorinemab, gosuranemab, Tilavonemab, and Zagotenemab. All four antibodies target the N-terminal region of tau protein but they showed a lack of efficacy in phase II clinical trials. Thus, now there is a transition towards developing antibodies that target other regions of the tau protein, such as the mid-domain, which may play a more vital role in the aggregation and propagation of tau pathology in AD. In AD, several facets of tau pathology could be addressed, as depicted in Figure 6.

- Tau aggregation inhibitors: The primary pathogenic species is currently thought to be oligomeric tau, which leads to acute toxicity in addition to defects in gene transcription, nuclear stability, mitochondrial health, neurotransmission, synaptic function, and protein degradation [180,181]. With the aim of inhibiting or reverting tau aggregation and restricting the spread of disease, multiple research groups have developed several small-molecule inhibitors.
  1. Methylene blue (MB) and leuco-methylthioninium bis(hydromethanesulphonate) (LMTM): Methylene blue (MB) and its reduced derivative leuco-methylthioninium bis(hydromethanesulphonate) (LMTM) [182,183] are among the compounds that have been explored thoroughly and have been shown to inhibit tau aggregation in vitro and in cells. It has been postulated that MB and LMTM primarily attach to Cys291 and Cys322 in tau’s microtubule-binding domain [184]. The effectiveness of LMTM has been evaluated in AD patients; nevertheless, the compound failed phase III clinical trials [185,186], indicating the need for more research on tau aggregation mechanisms.
  2. LMTX: A MB derivative called LMTX (also called TRx0237) can penetrate the blood–brain barrier (BBB) and has been shown to improve cognition and decrease tau aggregation in animal models. This medication has been the subject of multiple phase III trials in patients with mild-to-moderate AD, as well as an open-label extension study for the patients that finished the previous trials.
  3. Curcumin: Curcumin inhibits tau aggregation in vitro and reduces tauopathy in animal models [187]. The advantages of curcumin administration in MCI patients and healthy persons were investigated in a phase II clinical trial (NCT01383161). Those who took the medication showed improvements in their attention, visual memory, and long-term memory.
- Tau immunotherapies: The success of tau-targeted immunotherapies was reported in early studies [188,189] using vaccine and antibody approaches. Since then, multiple tau epitopes—including the N-terminus, mid-domain, microtubule-binding region, and several phospho-tau forms (e.g., p-tau202, p-tau231, p-tau396/404, p-tau409, p-tau422)—have been targeted and achieved promising preclinical and clinical outcomes [190,191]. Immunotherapy can work via extracellular or intracellular pathways, either active or passive. The benefits of active immunotherapy (administering a tau immunogen as a vaccine) include its low cost, potential to induce a polyclonal antibody response, and long-lasting effectiveness while the benefit of passive immunotherapy is its flexibility. Below, we discuss a few active and passive immunotherapies:
  1. E2814: An IgG1 antibody that attaches to extracellular tau and recognizes the HVPGG motifs in the tau microtubule-binding domain’s second and fourth repeats [192]. It has been documented that this antibody (or its mouse equivalent) inhibits tau seeding and aggregation in vitro, thereby reducing free tau containing the mid-domain in non-human primates, and attenuating the deposition of tau aggregates in mice injected with tau fibrils. In 2020, a phase I trial (NCT04231513) examined E2814’s immunogenicity, safety, and tolerability in healthy participants. Two participants acquired anti-E2814 antibodies, but no serious drug-related side effects were noted. The pharmacokinetics in the serum and CSF were proportional to the antibody dose, and the CSF exhibited a dose-related increase in antibody–tau associations that persisted for at least a month. The trial was widened to include a multiple-ascending-dose phase in 2021.
    E2814 was selected to be assessed in the Dominantly Inherited Alzheimer’s Network Trials Unit (DIAN-TU) preventive trial in 2021, whose participants have mutations in either presenilin or amyloid precursor protein. In order to recruit thirteen DIAN volunteers with mild-to-moderate cognitive impairment, a phase Ib/II experiment (NCT04971733) is being conducted. This experiment, which will continue until April 2025, will evaluate anti-drug antibodies, target engagement, the pharmacokinetics, safety, and tolerability. In DIAN patients with early-onset AD, additional phase II/III trials (NCT05269394 and NCT01760005) will evaluate the E2814 treatment either alone or in combination with anti-Aβ therapy (lecanemab). The safety, tolerability, biomarkers, and cognitive and other functional improvements induced by E2814, either by itself or in combination with lecanemab, will all be assessed in these trials. It is anticipated that both trials will finish in October 2027.
  2. Bepranemab: An IgG4 antibody called bepranemab (UCB0107) attaches itself to tau’s amino acids 235–250 in the vicinity of the microtubule-binding region. When tau was pre-incubated with the mouse version, it was discovered to prevent tau seeding in culture and in two mice models of tauopathy. Bepranemab’s pharmacokinetics, safety, and tolerability were assessed in three phase I trials. There were no anti-drug antibodies or drug-related safety concerns in the first study (NCT03464227) in healthy participants, and UCB0107 levels in the serum and CSF increased in a dose-dependent manner. The safety and pharmacokinetics were the main endpoints of a second phase I trial (NCT03605082), which also involved healthy participants. The findings have not yet been made public. The third phase I trial (NCT04185415) in PSP patients revealed no safety concerns. An ongoing phase II trial (NCT04867616) is evaluating its efficacy in patients with mild AD, with cognitive outcomes and tau PET imaging as the key endpoints; it is expected to conclude in 2025.
  3. BIIB080: To investigate the safety, tolerability, pharmacokinetics, and pharmacodynamics of tau ASO MAPTRx (also known as BIIB080) in individuals with mild AD, a phase Ib trial (NCT03186989) was started in 2017. The medication was said to be safe and able to decrease the levels of total tau (t-tau) and p-tau in the cerebrospinal fluid (CSF) in a dose-dependent manner during a press conference in 2021. Dose-dependent reductions in t-tau and p-tau in the CSF were demonstrated by additional phase I testing results that were reported in 2023 [193]. No appreciable improvements in cognitive, functional, behavioral, or neurological deficits were noted despite the decline in CSF measures.
    With cognitive alterations as the main objective, phase II testing has begun in individuals with mild cognitive impairment (MCI) brought on by AD or mild AD (NCT05399888) and will continue until December 2026.
  4. JNJ-63733657: JNJ-63733657 (NCT03375697) was tested in a phase I trial in both healthy people and patients with mild or prodromal AD [194] and no concerns about tolerability or safety were found. NCT03689153 and NCT05407818 are two further phase I trials that have finished that evaluated the pharmacokinetics, safety, and tolerability of JNJ-63733657 in healthy subjects. Additionally, phase II research (NCT04619420) is being conducted that will continue until 2025, evaluating the safety and effectiveness of, and tolerance to JNJ-63733657 in individuals with early-stage AD who have a positive tau PET scan. Cognitive change is the main outcome, with the safety, pharmacokinetics, brain tau load, CSF tau levels, and levels of other functional markers as secondary outcomes.
  5. LY3372689: In tauopathies, the O-GlcNAcylation of tau prevents tau phosphorylation and aggregation, which has protective effects. LY3372689 is an O-GlcNAcase (OGA) inhibitor that was demonstrated to be able to effectively enter the brain following a single dosage in rats; this study is currently being expanded to healthy humans, with LY3372689 demonstrating brain penetration and occupancy.

Table 3 lists all these tau-targeting drugs that are in clinical trials.

### 8.2. Targeting Neuroinflammation

In neurodegenerative illnesses, neuroinflammation is essential, and the activation of astrocytes and microglia contributes to the disease’s progression. Neuroinflammation therapies aim to alter immune responses without sacrificing their defense mechanisms [196]. Targeting specific inflammatory pathways, including blocking pro-inflammatory cytokines (IL-6, TNF-α) or adjusting microglial activation, are examples of the emerging tactics [179,197]. Anti-inflammatory drugs are screened using organotypic brain-slice cultures and glial cell culture models. Furthermore, medications that block microglial activation, such as minocycline, have demonstrated potential in reducing neuroinflammation [198]. However, challenges in translating these findings to clinical applications persist, as neuroinflammation is a double-edged sword that can be both protective and detrimental.

### 8.3. Targeting Tau Phosphorylation

AD and other tauopathies are characterized by the production of neurofibrillary tangles, which are caused by hyperphosphorylation of tau protein. The goals of therapeutic approaches are to stabilize microtubules, encourage the removal of tau, or prevent tau phosphorylation. It has been demonstrated that kinase inhibitors that target cyclin-dependent kinase 5 (CDK5) and glycogen synthase kinase-3 (GSK-3) can decrease tau phosphorylation [61,199,200]. The GSK-3 inhibitor lithium has been studied for its potential neuroprotective benefits [201]. Furthermore, epothilone D and other microtubule-stabilizing drugs improve microtubule integrity and stop tau-induced toxicity [202]. The goal of immunotherapies that target phosphorylated tau species is to aid in its removal. Clinical trials are presently underway for a few monoclonal antibodies that target tau clumps [203]. The development of tau-based treatments is still difficult, though, because tau pathology frequently manifests late in the course of the disease, making early intervention essential.

### 8.4. Targeting Oxidative Stress

Oxidative stress damages proteins, DNA, and lipids, which leads to neurodegeneration. Antioxidant-based treatments aim to restore mitochondrial activity and lower reactive oxygen species (ROS) [204]. The neuroprotective qualities of substances, including vitamin E, coenzyme Q10, and N-acetylcysteine, have been investigated. In preclinical studies, polyphenols such as resveratrol and curcumin have demonstrated promise in lowering oxidative damage [205,206]. Antioxidants that target mitochondria, like MitoQ, are made to reside inside mitochondria and stop oxidative stress from damaging neurons. Another possible strategy is strengthening endogenous antioxidant pathways, such as the Nrf2-ARE pathway [207]. Clinical trials have produced inconsistent results despite encouraging results in animal models. This is probably because of the limited bioavailability and difficulties in addressing oxidative damage at phases relevant to the disease.

### 8.5. TargetingAβ

One of the main pathogenic characteristics of AD is Aβ buildup. Treatment approaches focus on the synthesis, aggregation, and removal of Aβ. The goal of β-secretase (BACE) and γ-secretase inhibitors is to stop the production of Aβ. However, many have failed in clinical trials because they are toxic or ineffective [208,209]. Aβ plaques are targeted for removal by immunotherapy employing monoclonal antibodies, such as aducanumab [210]. Although some have demonstrated cognitive benefits, adverse inflammation-related problems are still a worry. Other strategies include employing small compounds to stop Aβ oligomerization and increase Aβ breakdown using proteolytic enzymes like neprilysin. Targeting Aβ has not yet resulted in a permanent treatment for AD despite tremendous efforts, underscoring the necessity of multiple methods.

Recent advancements in aging and AD research have resulted in the discovery of novel therapeutic targets that are beyond classical Aβ and tau approaches. According to Zhu et al. (2015), dasatinib and quercetin together diminish the pro-inflammatory senescence-associated secretory phenotype (SASP) and specifically kill senescent cells, which alleviates tissue dysfunction, particularly in the brain [211]. Additional approaches for combating age-associated proteinopathy involve substances that regulate mitochondrial biogenesis (for example, through PGC-1α pathways) and autophagy inducers, including rapamycin analogs [212]. Additionally, there are now clinical trials for innate immune modulators such AL002, a monoclonal antibody that targets TREM2 to modulate microglial function [213]. ALZ-801, a prodrug of tramiprosate, is an oral, small-molecule inhibitor of Aβ oligomer formation in clinical development for AD. It is particularly targeted at individuals carrying two copies of the apolipoprotein E ε4 allele (APOE4/4 homozygotes), who are at a significantly higher risk for developing AD. These strategies highlight the trend toward multitargeted, age-adapted therapeutic design in AD.

## 9. Conclusions

Effective therapeutic strategies are still elusive despite the tremendous progress in understanding the mechanisms causing age-related neurodegeneration, especially in AD. There is currently no proven cure or preventive treatment, despite several treatments being investigated in current research, such as those targeting protein aggregation, oxidative stress, neuroinflammation, and Aβ pathology. The absence of highly efficient disease models that faithfully mimic human neurodegeneration remains a key obstacle. Age is the biggest risk factor for neurodegenerative illnesses, as this research has shown, underscoring the complex link between AD and aging. The pathogenic mechanisms underlying AD include aging-related hallmarks such as cellular senescence, chronic inflammation, and mitochondrial failure.

Although comprehending these systems may open up new intervention options, it is still very difficult to turn these discoveries into clinically effective therapies. To facilitate prompt intervention, future research should concentrate on improving disease models, creating multi-targeted therapy strategies, and identifying early biomarkers. Furthermore, using developments in genomics and precision medicine could open the door to more individualized approaches to the fight against AD. The ultimate objective is still to create disease-modifying therapeutics that can postpone or completely prevent neurodegeneration while using the existing treatments to provide symptomatic alleviation. Resolving these issues will enhance the aging population’s health and quality of life.

## Figures and Tables

**Table 1:** Differences in various features of normal aging brains and AD brains.

No	Features		Normal Aging Brain	AD Brain	Refs.
1.	Structural differences	a. Brain size	Mild to low shrinkage	Severe shrinkage observed in hippocampus	[27,28,29,30]
		b. Neurodegeneration	Low	Very severe loss of neurons	
		c. Ventricles	Slightly enlarged	Significantly enlarged	
		d. Cortical atrophy	Mild	Extensive	
2.	Cellular and molecular differences	a. Aβ plaques	Minimal	Large extracellular deposits	[31,32,33,34,35,36,37]
		b. NFTs	Few	Abundant	
		c. Neuroinflammation	Mild, associated with aging	Chronic inflammation and neuronal damage	
		d. Tau phosphorylation	Basal level	Highly phosphorylated	
		e. Synaptic loss	Neural plasticity intact	Severe synapse loss and impaired communication between neurons	
3.	Cognitive differences	a. Memory decline	Forgetfulness	Severe memory loss	[28,38,39,40,41,42]
		b. Cognitive function	Slower processing but intact reasoning	Impaired reasoning and confusion	
		c. Communication	Occasionally forgets words but remembers later	Struggles with word-finding, conversation, and understanding language	
		d. Behavioral changes	Mild mood changes	Significant mood swings, aggression, apathy, and social withdrawal	

**Table 2:** Tau phosphorylation sites and related kinases.

Tau Phosphorylating Kinase	Site(s) of Phosphorylation	References
GSK-3β	Ser199, Ser202, Ser396, Ser404, Ser422, Thr205, Thr212, Thr217, Thr231	[120,123,124,125,126,127,128,129,130,131,132,133,134,135,136,137]
CaMK-II	Ser416	
CDK5	Ser202, Ser396, Ser404, Ser422, Thr205, Thr212, Thr217, Thr231	
MARK4	Ser262	
PKA	Ser409	
DYRK-1	Thr212	
MAPKs	Ser202, Thr231	

**Table 3:** Tau-targeting drugs in clinical trials. Adapted from [195].

Agent	Therapeutic Purpose	Target	Mechanism of Action	Clinical Trial	Start Date	Estimated Primary Completion Date
Methylene Blue	Disease-modifying small molecule	Tau	Tau protein aggregation inhibitor	NCT02380573	July 2015	April 2022
E2814	Disease-modifying biologic	Tau	Anti-tau monoclonal antibody	NCT01760005 NCT05269394	December 2012 December 2021	October 2027 July 2027
Bepranemab	Disease-modifying biologic	Tau	Anti-tau monoclonal antibody that binds to the central region of tau	NCT04867616	June 2021	May 2024
BIIB080	Disease-modifying biologic	Tau	Antisense oligonucleotide that inhibits the translation of tau mRNA into the tau protein	NCT05399888	August 2022	November 2027
JNJ-63733657	Disease-modifying biologic	Tau	Monoclonal antibody targeting soluble tau (mid-region of tau)	NCT04619420	January 2021	March 2025
LY3372689	Disease-modifying small molecule	Tau	O-GlcNAcase enzyme inhibitor	NCT05063539	September 2021	July 2024

## Data Availability

No data was used for the research described in the article.
